# Quantification of Macular Carotenoids over a Wide Dynamic Range in Plant Matrices and Caco-2 Cells Using a Single Transferable Analytical Method

**DOI:** 10.3390/foods15060981

**Published:** 2026-03-10

**Authors:** Jenani Sutharsan, Lewis Adler, Alison Jones, Jayashree Arcot

**Affiliations:** 1 Food and Health Group, School of Chemical Engineering, University of New South Wales, Sydney, NSW 2052, Australia; 2 Bioanalytical Mass Spectrometry Facility, Mark Wainwright Analytical Centre, University of New South Wales, Sydney, NSW 2052, Australia; 3 Food Science, School of Life and Environmental Sciences, University of Sydney, Sydney, NSW 2050, Australia

**Keywords:** lutein, zeaxanthin, liquid chromatography, in vitro digestion, Caco-2 cells, bio-accessibility

## Abstract

Lutein and zeaxanthin are macular carotenoids known for their protective role against major eye diseases. The bio-accessibility of these macular carotenoids is extremely low, with a limited amount synthesised in plants. Quantifying these compounds in plants/biological samples is challenging because of their structural similarity. Although numerous methods have been reported for quantifying macular carotenoids, there is currently no unified chromatographic technique that can be applied for the separation and quantification of these carotenoids across diverse matrices over a broad dynamic range while also incorporating an effective extraction step. Biochemical processes during digestion and absorption further lower carotenoid levels in the body (bioavailability), making precise measurement of their esterified forms necessary. Here, we incorporate an alkaline hydrolysis extraction and present a single liquid chromatographic method applicable to both PDA and MS detection for the separation and quantification of lutein and zeaxanthin across various matrices (food, digesta, and Caco-2 cells) and concentration ranges. It utilises common solvents for the mobile phase system and a C30 column. The reverse-phase method achieved excellent recoveries in spiked samples, acceptable relative standard deviations (RSDs) for validation parameters, and offers potential for high-throughput analysis while being transferable between matrices (from plant to Caco-2 cells).

## 1. Introduction

Lutein, zeaxanthin and meso-zeaxanthin are oxygenated carotenoids (xanthophyls). They are collectively called macular pigments (MPs) due to their presence in the retina at higher concentrations [1,2,3]. Epidemiological studies have reported a positive correlation between a higher intake of these MPs and a reduced incidence of age-related macular degeneration, cataract and retinitis pigmentosa [4,5,6]. Lutein (3R,3R,6R-β, ε-caroten-3,3-diol) frequently co-occurs in biological systems with its configurational stereoisomer, zeaxanthin (3R,3R-β,β-caroten-3,3-diol), differing in the position of the ε- versus β-ionone ring. Lutein has 10 conjugated double bonds (nine conjugated double bonds in the polyene chain and a single double bond in the b-ionone ring), whereas zeaxanthin contains 11 conjugated double bonds (nine conjugated double bonds in the polyene chain and two double bonds in the b-ionone ring) [7]. These carotenoids cannot be synthesised by the human body; hence, dietary sources are essential [8]. Green leafy vegetables such as kale, spinach, broccoli and corn are rich sources of these xanthophylls [9]. Generally, the ratio of lutein and zeaxanthin contents in most foods is 5:1 [10]. A few foods, such as orange pepper, red pepper and hot chilli, have higher zeaxanthin than lutein [11]. There is a need for accurate quantification of these MPs in foods and biological samples for various reasons, such as to understand the function of individual MPs, to consume the right amount of food and to prepare supplements with the correct ratio of these compounds. However, their structural similarity and their presence at low levels in food make the quantification of these pigments a challenging task [11].

A variety of analytical techniques (colourimetric, spectrophotometric, fluorometric, paper, open-column and thin-layer chromatography, high-performance liquid chromatography (HPLC), and capillary electrophoresis) have been applied for the identification and quantification of carotenoids in food matrices. However, for structural characterisation and compound confirmation, HPLC is commonly coupled with NMR spectroscopy and mass spectrometry. Among the available techniques, HPLC is considered the gold standard for the separation and analysis of carotenoids in biological and food samples, as it effectively resolves cis-isomers from their all-trans counterparts [12,13]. These chromatographic methods have employed both C18 and C30 columns with various mobile phase systems, encompassing both HPLC and LC–MS platforms [14,15,16,17,18,19,20] (Table S1). However, compared to C18 columns, C30 columns offer superior resolution for carotenoids with similar polarity and are therefore generally preferred for separating geometrical isomers [14,21,22]. Lutein and zeaxanthin were quantified in marigold and poultry feed using a C18 column on an HPLC and UHPLC with the same mobile phase used in the present study. However, the limit of detection of the present study is lower than that of the previous method [23].

Moreover, most reported methods did not include a purification or saponification step prior to extracting these xanthophylls from the samples. Saponification, or alkaline hydrolysis, is recommended for the accurate quantification of xanthophylls, as they commonly occur in esterified forms with fatty acids in fruits and vegetables due to the presence of hydroxyl groups in their structure [21]. In addition, saponification degrades lipids, chlorophyll, and other matrix constituents that may interfere with chromatographic analysis [12].

Bernstein et al. [24] quantified macular carotenoids from tissues of the human eye using silica-based nitrile-bonded and C18 columns. The solvents used were hexane, dichloromethane, acetonitrile and methanol. A few other studies have reported the use of acetonitrile:methanol:dichloromethane (either 6:2:2 or 7:1:2) with a C18 (ODS) column and a C30 column [25,26]. Reproducibility, however, was unachievable due to co-elution with the preparation and samples employed in this study. Acetone and water were used by [19] for the separation of lutein and zeaxanthin. Aetone may not be compatible with many chromatographic systems, especially those using plastic tubing. In another study, hexane and isopropanol (90:10) were used by [27] with a Daicel Chiralpak IA-3 column, which is not available in many laboratories due to its high cost and incompatibility with water from samples.

Mass spectrometry is another approach to accurately quantify these MPs from biological matrices, where the MPs are present at very low levels and have high potential for spectral interferences. In a previous study on the separation of lutein and zeaxanthin from a standard mix of other carotenoids, LC-MS was performed in the positive ion mode using a TurboISP-MS system [28]. Two ODS Hypersil columns were used with an isocratic solvent system consisting of acetonitrile, methanol and dichloromethane at the ratio of (71:22:7). However, the positive ion mode may not be sensitive enough to overcome the spectral interferences encountered with the biological samples (matrices) [29].

Most of the aforementioned methods did not include extractions by either saponification or alkaline hydrolysis methods. Ref. [30] has reported an alkaline hydrolysis extraction method for fruits and vegetables, followed by solvent extraction. This step is considered essential for simplifying separation, as it eliminates substances such as chlorophylls and lipids, reduces analytical interferences and enhances recoveries [31,32].

Given the limitations of currently available chromatography methods suitable for the extraction methods used, and the lack of a single (unified) validated method for the separation and quantification of lutein and zeaxanthin from various matrices across a wide dynamic range (six orders of magnitude), this study aimed to develop a single (unified), robust, and validated method. This proposed method, coupled with alkaline hydrolysis, is designed to separate and quantify lutein and zeaxanthin from diverse matrices (food, digesta and in vitro cellular uptake) across a broad range of concentrations (detection limits) within a short run time of 15 min, enabling higher sample throughput. In addition, applying the same procedure across fresh food to digesta and in vitro cellular contents (Caco-2 cells) allows for a more appropriate comparison of the bio-accessibility of these xanthophylls. The proposed method was validated according to “The International Guidelines for Analytical Techniques Validation” [16]. To test the applicability of this method on food and other matrices, the separation and quantification of lutein and zeaxanthin using both HPLC and LC-MS were performed on both a standard mix, selected fresh vegetables, their micellar fractions obtained after in vitro digestion and their cellular uptake by Caco-2 cells.

## 2. Materials and Method

### 2.1. Materials

Fresh orange pepper, cauliflower, kale and watermelon were purchased from a local supermarket (Sydney, Australia). The lutein (Supelco, Bellefonte, PA, USA, 07168-1MG), zeaxanthin (Supelco, 04681-1MG-F) and trans-β-apo-8′-carotenal (1107-26-2) standards were purchased from Sigma Aldrich (Sydney, Australia). The acetinitrile, methanol, hexane, di-ethyl ether and ethyl alcohol (Labscan) used for the extraction and quantification were bought from the university chemical store (UNSW, Australia) and all the solvents used were of HPLC grade.

### 2.2. In Vitro Digestion

Fresh kale (*Brassica oleracea*) (10 g) was first subjected to in vitro digestion following a method reported by [32]. Briefly, kale with 4.5 mL of salivary fluid and 0.5 mL of α-amylase solution (2000 U/mL) was incubated at 37 °C by shaking at 100 rpm for 10 min to simulate oral digestion. Then, the pH was adjusted to 2.0 using 4 M HCl, and 12 mL of gastric solution was added to initiate gastric digestion, and the samples were incubated again at 37 °C with shaking at 100 rpm for 60 min. At the end of the gastric phase, the pH was raised to 5.5 using 1 M NaHCO_3_ before adding 6 mL of duodenal solution to commence the intestinal phase. The pH was further adjusted to 7.0 with 1 M NaOH, and the total volume was brought to 50 mL with 0.15 M NaCl. Intestinal digestion proceeded for 2 h at 37 °C with shaking at 100 rpm.

At the end of the digestion process, the digesta was centrifuged at 4000× *g* for 20 min and subsequently filtered through the cellulose ester membrane (0.65 µm, MF-Millipore^TM^ membrane filter, Merck KGaA, Darmstadt, Germany) to concentrate the xanthophylls from the supernatant. The micellar fraction was used for the uptake study with Caco-2 cells.

### 2.3. Cellular Uptake Study

Human carcinogenic colon Caco-2 cells were used for the cellular experiments. The cells were grown on Dulbecco’s modified Eagle medium (DMEM, Gibco, Thermo Fisher Scientific, Victoria, Australia) supplemented with 10% foetal bovine serum (Invitrogen, Thermo Fisher Scientific, Australia), 1% non-essential amino acid (Gibco), 1% GlutaMax™ (Gibco), and 1% penicillin and streptomycin (Sigma Aldrich).

They were maintained on 75 cm^2^ plastic flasks (Corning^®^, Corning Inc., Berlin, Germany) in a CO_2_ incubator (Touch 190S, LEEC, Netherfield, UK) at 37 °C and 5% CO_2_ and sub-cultured when they reached 80% confluence. For the cellular uptake, antioxidant and anti-inflammatory studies, cells at passages from 50 to 55 were used.

For the cellular uptake experiments, Caco-2 cells were seeded on 6-well microtiter plates at a cell density of 2 × 10^5^ cells/mL and grown for 14 days with fresh medium replacements on alternative days. Then they were treated with the filtered kale digesta, with a digesta:growth medium ratio of 1:3 and incubated at 37 °C. After 4 h, the medium was removed, and the cells were washed with Dulbecco phosphate-buffered saline (DPBS). Then they were collected with Milli Q water and stored at −80 °C until further analysis [33].

### 2.4. Extraction of Samples for the Chromatographic Analysis

Edible portions of freeze-dried kale, orange pepper, cauliflower and watermelon (0.5 g) were extracted using an alkaline hydrolysis and a solvent extraction method outlined by [30] with slight modifications. Briefly, the fresh samples (2 g), digesta (10 mL) and Caco-2 cells (3 mL) were hydrolysed with 2 mL of potassium hydroxide (60 g/L), 3 mL of ethanol (95%), 1 mL of sodium chloride (10 g/L), and 5 mL of ethanolic pyrogallol (60 g/L) in a water bath at 50 °C for 30 min. After hydrolysis, the samples were cooled in an ice bath and an additional 15 mL of sodium chloride (10 g/L) was added. Then, the samples were extracted with 30 mL of n-hexane and ethyl acetate mixture (9:1) until the organic layer became colourless. Trans-β-apo-8′-carotenal was used as the internal standard. Then, the pooled organic layer was dried under nitrogen, reconstituted with 300 µL of the mobile phase (acetonitrile: methanol: ammonium acetate (0.3 g/L) in the ratio of 95:4.5:0.5 and filtered through a 0.22 µm PTFE membrane (Kinesis, Redland Bay, Australia) before the HPLC or LCMS analysis.

### 2.5. Standard Preparation

Stock standards of lutein and zeaxanthin were prepared in tetrahydrofuran (1 mg/mL) and were stored under a nitrogen blanket at −80 °C. The concentrations of lutein and zeaxanthin were confirmed before analysis on the UV-VIS spectrophotometer (UV-1800, series spectrophotometer, Shimadzu, Japan) at 454 nm (extinction coefficient = 134,400 L/mol.cm^−1^). The working standards were freshly prepared by serial dilution in ethyl alcohol.

### 2.6. Separation of Lutein and Zeaxanthin on HPLC

Both the lutein and zeaxanthin from the fresh vegetables and the micellar fractions after the in vitro digestion were identified and quantified separately on a reversed phase high-performance liquid chromatographic (HPLC) system (LC-20AD, Shimadzu, Tokyo, Japan) coupled with a photodiode array detector (PDA; (SPD-M20A), an auto sampler (SIL-30AC), degasser (DGU-20A_5_), column oven (CTO-20A) and a communications bus module (CBM-20A). Separation of the lutein and zeaxanthin was carried out using an Acclaim^TM^ C30 column (2.1 × 250 mm, 3 µm particle size, Thermo Fisher Scientific Inc., USA) with an isocratic flow rate of 0.5 mL/min at the column temperature of 25 °C. The injection volume was 5 µL and the total run time was 15 min per sample.

Acetonitrile: methanol: ammonium acetate (0.3 g/L) at a ratio of 95:4.5:0.5 was used as the mobile phase. It was filtered and sonicated prior to use. Methanol (10%) was used as a wash solution for the needle.

### 2.7. Separation of Lutein and Zeaxanthin on LC-MS

Extracts from the Caco-2 cells and digests of kale were analysed for lutein and zeaxanthin contents using two types of mass spectrometers. The first system is the Ultimate 3000, with a column oven injector and in-line Accela PDA (Thermo Fisher Scientific Inc., Waltham, MA, USA) interfaced with a Hybrid Ion trap-Orbitrap mass spectrometer (LTQ Orbitrap XL™, Thermo Fisher Scientific Inc., USA), and the second system consisted of a Vanquish UHPLC system coupled with a Q-Exactive Plus mass spectrometer (ThermoFisher Scientific). Samples were analysed in negative mode atmospheric pressure chemical ionisation.

The mass spectrometer was equipped with an atmospheric pressure chemical ionisation probe (API). The APCI source working parameters were optimised and involved a vaporiser temperature of 320 °C, sheath gas flow rate of 80 arbitrary units (au), auxiliary gas flow rate of 5 au, sweep gas flow rate of 0 au, discharge current of 20 µA, capillary temperature of 275 °C, capillary voltage of −2 V, and tube lens voltage of −68 V. A *m*/*z* scan range of 400–580 was set. Lutein and zeaxanthin were separated in a negative ion Fourier Transformation mode at a resolution of 60,000.

The Q-Exactive Plus mass spectrometer was operated in the data-dependent analysis mode—automatically acquiring MS/MS data. The instrument was scanned from 400 to 800 at a resolution of 140 K, with MS/MS of the top 5 ions at 35 K. The source conditions were spray current 4 (negative), sheath gas 40 au, auxiliary gas 10 au. The probe heater temperature was 358 °C and the capillary temperature was 250 °C. S-Lens was 50 V. The instrument was calibrated immediately prior to data acquisition. Samples were run in duplicate, and the data analysis was performed using FreeStyle software (ThermoFisher).

For both mass spectrometers, an Acclaim^TM^ C30 column (2.1 × 250 mm, 3 µm particle size, Thermo Fisher Scientific Inc., USA) was used for the measurement of carotenoids with an isocratic flow rate of 0.5 mL/min at 25 °C. The mobile phase was acetonitrile: methanol: ammonium acetate (0.3 g/L) in a ratio of 95: 4.5: 0.5. The injection volume was 5 µL.

### 2.8. Method Validation

Validation of this method on the HPLC and LC-MS was carried out with respect to linearity, limits of detection (LOD), limits of quantification (LOQ), instrumental precision and intra-day and inter-day precision. Linearity was measured with a series of mixed standards of lutein and zeaxanthin covering the entire working range (0.5 ng–100 µg) of concentrations on both HPLC and LC-MS. Each concentration was run in triplicate, and the mean values were used to construct the calibration curve to get the linearity equation and the respective correlation coefficient. Instrument precision was studied by injecting the same extract seven times, whereas intra-day and inter-day precision were evaluated by analysing an extract five times on three consecutive days. The LODs were assessed by considering the quantities that produced a signal of peak height 3.3 times the size of the background noise, while LOQs were assessed as a signal peak height 10 times the size of the background noise.

## 3. Results and Discussion

### 3.1. Linearity, Limit of Detection and Limit of Quantification

**Figure 1:**
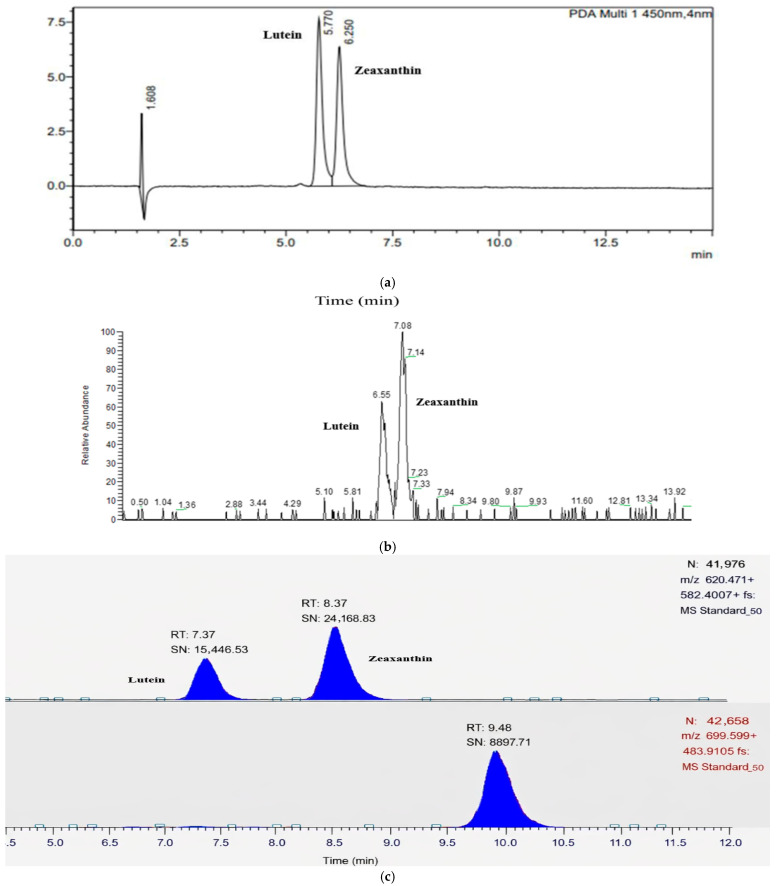
Separation of lutein and zeaxanthin from a standard mix using HPLC (**a**); separation of lutein and zeaxanthin from a standard mix using LTQ-Orbitrap (**b**); and separation of lutein and zeaxanthin from a standard mix using QE Plus MS (**c**), where A, B, C are lutein, zeaxanthin and trans-β-apo-8′-carotenal, respectively.

The linearity and sensitivity parameters for both the HPLC and LC-MS analysis are reported in Table 1 and Table 2, and the chromatograms of the separation of lutein and zeaxanthin on both HPLC and LC-MS from a standard mix are shown in Figure 1.

The peaks of both compounds were sharp and symmetrical with no co-elution of the isomers. The stationary phase (e.g., column properties) and the composition of the mobile phase are the two major factors that affect the resolution of sample separation [23]. In the proposed method, the column particle size of 3 µm would have facilitated better separation of lutein and zeaxanthin within a short period of time (<7 min) compared to the other methods, where a column with a particle size of 5 µm is used [19,25,30,34].

The calibration curves (Figures S1 and S2, Supplementary Materials) were constructed by plotting the peak area against the concentrations of the mixed lutein and zeaxanthin standards. The linearity was evaluated by injecting a triplicate of a minimum of seven concentration levels of the standard mix. A satisfactory linearity was obtained with the higher coefficient of determination of above 99% for both compounds using both HPLC and LC-MS analysis.

The lower LOD and LOQ values obtained for the LC-MS (Table 1a,b) show better sensitivity compared to the analysis on HPLC. Moreover, the LOD and LOQ obtained in this method for HPLC are lower than the values in the previously reported methods [16,23,35].

The LOD and LOQ values obtained by the proposed method on LC-MS are lower than the previously reported values (Table S2).

### 3.2. Validation of the Proposed Method

The validation parameters of the new method using both HPLC and LC-MS are summarised in Table 2, Table 3 and Table 4. According to the RSD values (<3%) obtained for each compound when injected five times on the same day, both chromatographic systems produced a satisfactory response in terms of peak area and retention times. These values are consistent with the reported results in the literature [16,23]. The instrument precision for the peak area and the retention time of zeaxanthin is higher in HPLC compared to that of LC-MS (details presented in Tables S1–S4, Supplementary Materials).

Fresh kale and Caco-2 cell extracts were spiked with a standard mix of lutein and zeaxanthin, and the results are reported in Table 5. A recovery percentage of ≥90.5% for both compounds on both chromatography systems demonstrates the efficiency of the method for the separation and quantification of lutein and zeaxanthin. The details of this analysis are summarised in Table S5 of the Supplementary Materials.

### 3.3. Application of the Method to the Food and Biological Samples

The validated method was applied to fresh kale and digesta after in vitro digestion and Caco-2 extracts after the uptake study with the kale digesta. The lutein and zeaxanthin contents of the fresh kale and the digesta were analysed on the HPLC, whereas the cell extracts after absorption/uptake were analysed on the LC-MS because of the very low concentrations of the compounds in the extracts (Table 5).

The lutein content of kale was 420 (µg/g DM) and this value is consistent with the previously reported values [6,36,37,38]. The zeaxanthin content of kale quantified in this method was 34.35 (µg/g DM); however, there are no reported measurements of zeaxanthin for kale for comparison. Similarly, there were no data available in the literature for the lutein and zeaxanthin uptake of Caco-2 cells from kale.

However, the uptake values obtained using the newly developed method in this study are approximately similar (1.16 µg/100 g fresh weight) to the uptake of lutein by the Caco-2 cells from spinach obtained in a previous study [33].

**Figure 2:**
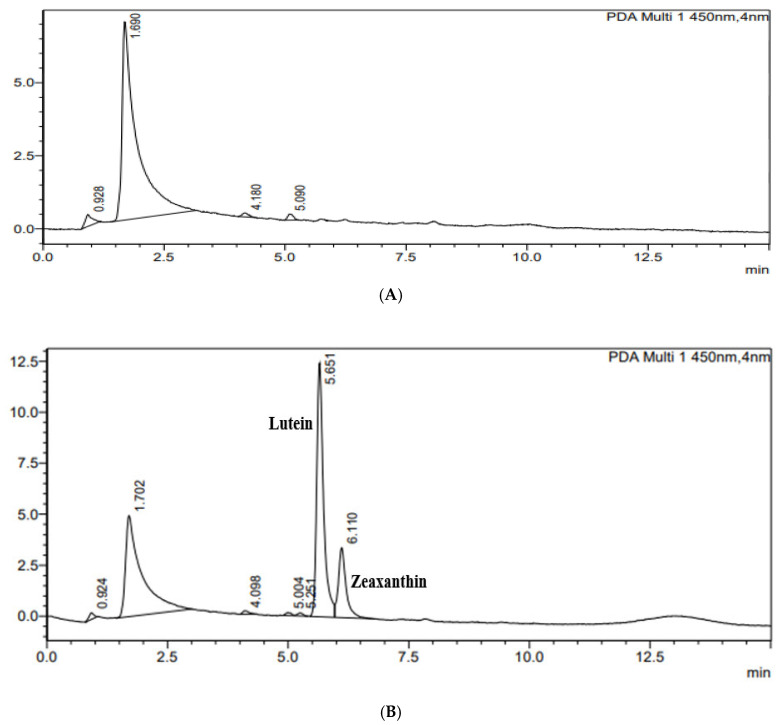
(**A**,**B**) are HPLC chromatograms of fresh watermelon and fresh watermelon spiked with standard mix of lutein and zeaxanthin, respectively.

The same method was also applied to watermelon. According to the literature, watermelon does not have either lutein or zeaxanthin [38,39]. Extracts of watermelon with and without the addition of the standard mix containing lutein and zeaxanthin were analysed and the corresponding chromatograms are shown in Figure 2A,B. As expected, this proposed method did not show lutein and zeaxanthin in those samples.

Figure S7A shows the separation of lutein and zeaxanthin from kale, wherein the lutein content is higher than the zeaxanthin content. Whereas, in orange pepper, the zeaxanthin content is higher than lutein content (Figure S7B).

## 4. Conclusions

A single (unified) method is proposed for the quantification of lutein and zeaxanthin in both food and biological matrices, using HPLC and LC-MS in combination with an extraction procedure. It utilises common solvents for the mobile phase system and a C30 column. The alkaline hydrolysis extraction coupled with reversed-phase separation gave reproducible results consistently for the analysed samples, even with potential spectral interferences from micellar fractions and cell matrices. Good separation and resolution were obtained for these compounds within 7 min of elution, with efficient sample throughput as the total run time is only 15 min. The spike recovery of above 90% and the lower RSD % for the instrument precision for both chromatographic systems demonstrate the efficiency of the method. The proposed method can be routinely used for the quantification of lutein and zeaxanthin in food and pharmaceutical products as well as biological matrices.

## Figures and Tables

**Table 1:** (**a**) Sensitivity and linearity characteristics of lutein and zeaxanthin measured using HPLC. (**b**) Sensitivity and linearity characteristics of lutein and zeaxanthin measured on LC—MS.

(**a**)					
	**Linear Range** **(µg/mL)**	**Regression Equation**	**Coefficient of Determination**	**LOD** **(µg/mL)**	**LOQ (µg/mL)**
Lutein	0.05–100	Y = 8247.5X − 1836.5	0.9976	0.03	0.12
Zeaxanthin	0.05–100	Y = 12,825X − 14,085	0.9980	0.03	0.12
(**b**)					
	**Linear Range** **(ng/mL)**	**Regression Equation**	**Coefficient**	**LOD** **(ng/mL)**	**LOQ (ng/mL)**
Lutein	0.5–1	Y = 2 × 10^8^X − 2 × 10^6^	0.9980	0.40	2.10
Zeaxanthin	0.5–1	Y = 3 × 10^8^X + 1 × 10^6^	0.9990	0.40	2.10

LOD—limit of detection; LOQ—limit of quantification. In the regression equation y = ax + b, x refers to the concentration ratio of lutein and zeaxanthin and y refers to the peak area.

**Table 2:** Method validation parameters for HPLC.

Instrumental Parameters	Lutein		Zeaxanthin	
	Peak Area (RSD %)	Retention Time (RSD %)	Peak Area (RSD %)	Retention Time (RSD %)
Instrumental precision	0.60	0.30	0.10	0.10
Intra-day precision	1.60	0.60	2.50	0.20
Inter-day precision	0.10	0.20	2.00	1.00

**Table 3:** Method validation parameters for LC-MS (LTQ-Orbitrap).

Instrumental Parameters	Lutein		Zeaxanthin	
	Peak Area (RSD %)	Retention Time (RSD %)	Peak Area (RSD %)	Retention Time (RSD %)
Instrumental precision	0.96	0.01	0.50	0.50
Intra-day precision	0.70	0.40	0.60	0.60
Inter-day precision	2.30	0.60	1.10	2.10

**Table 4:** Recoveries (%) of lutein and zeaxanthin from kale on HPLC and Caco-2 cell extracts on LC-MS (LTQ-Orbitrap).

	From Kale on HPLC		Caco-2 Extracts on LC-MS	
	Recovery	RSD	Recovery	RSD
Lutein	99.28 ± 0.59	0.60	91.20 ± 1.6	1.80
Zeaxanthin	97.80 ± 0.82	0.85	90.73 ± 0.67	0.74

**Table 5:** Mean lutein and zeaxanthin contents (µg/g DM) of fresh kale, micellar fractions and cellular uptake.

Kale	Lutein	Zeaxanthin
Fresh kale	420.44 ± 3.22	34.35 ± 2.08
Micellar fraction	197.70 ± 0.34	12.74 ± 0.12
Cellular uptake	1.09 ± 0.14	0.74 ± 0.08

All values are means of duplicate extractions and triplicate determinations.

## Data Availability

The original contributions presented in this study are included in the article/Supplementary Material. Further inquiries can be directed to the corresponding author.

## Supplementary Materials

The following supporting information can be downloaded at https://www.mdpi.com/article/10.3390/foods15060981/s1, Figure S1: A and B are linearity curves of lutein and zeaxanthin on HPLC, respectively; Figure S2: C and D are linearity curves of lutein and zeaxanthin on LC-MS, respectively; Figure S3: Separation of lutein and zeaxanthin from a mix standard on HPLC; Figure S4: Separation of lutein and zeaxanthin from a mix standard on LC-MS QE Plus; Figure S5: Separation of lutein and zeaxanthin from kale digest on LC-MS QE Plus; Figure S6: Separation of Lutein and zeaxanthin from kale digesta on LTQ-Orbitrap; Figure S7: A; HPLC chromatogram of fresh kale, B; HPLC chromatogram of fresh orange pepper; Table S1: Summary of the generally followed methods reported over the past 10 years for the separation of lutein and zeaxanthin from various samples; Table S2: The LOD and LOQ values from the previously validated methods to separate lutein and zeaxanthin; Table S3: HPLC Instrumental precision (seven consecutive injections of the same extract); Table S4: ILC-MS Instrumental precision (Seven consecutive injections of the same extract); Table S5: Intra-day and inter day precision (5 extracts were prepared and injected for 3 days) on HPLC; Table S6: Intra-day and inter day precision (5 extracts were prepared and injected for 3 days) on LC-MS; Table S7: Results of spike samples of kale on HPLC; Table S8: Results of spike samples of Caco-2 cell extracts on LC-MS.

## References

[1] Perry A., Rasmussen H., Johnson E.J. (2009). Xanthophyll (lutein, zeaxanthin) content in fruits, vegetables and corn and egg products. J. Food Compos. Anal..

[2] Loughman J., Loskutova E., Butler J.S., Siah W.F., O’Brien C. (2021). Macular Pigment Response to Lutein, Zeaxanthin, and Meso-zeaxanthin Supplementation in Open-Angle Glaucoma: A Randomized Controlled Trial. Ophthalmol. Sci..

[3] Nolan J.M., Meagher K., Kashani S., Beatty S. (2013). What is meso-zeaxanthin, and where does it come from?. Eye.

[4] Panova I.G., Yakovleva M.A., Tatikolov A.S., Kononikhin A.S., Feldman T.B., Poltavtseva R.A., Nikolaev E.N., Sukhikh G.T., Ostrovsky M.A. (2017). Lutein and its oxidized forms in eye structures throughout prenatal human development. Exp. Eye Res..

[5] Ziegler J.U., Wahl S., Würschum T., Longin C.F.H., Carle R., Schweiggert R.M. (2015). Lutein and Lutein Esters in Whole Grain Flours Made from 75 Genotypes of 5 Triticum Species Grown at Multiple Sites. J. Agric. Food Chem..

[6] De Azevedo C.H., Rodriguez-Amaya D.B. (2005). Carotenoid composition of kale as influenced by maturity, season and minimal processing. J. Sci. Food Agric..

[7] Nwachukwu I.D., Udenigwe C.C., Aluko R.E. (2016). Lutein and zeaxanthin: Production technology, bioavailability, mechanisms of action, visual function, and health claim status. Trends Food Sci. Technol..

[8] Britton G. (2020). Carotenoid research: History and new perspectives for chemistry in biological systems. Biochim. Biophys. Acta (BBA)-Mol. Cell Biol. Lipids.

[9] Zafar J., Aqeel A., Shah F.I., Ehsan N., Gohar U.F., Moga M.A., Festila D., Ciurea C., Irimie M., Chicea R. (2021). Biochemical and immunological implications of lutein and zeaxanthin. Int. J. Mol. Sci..

[10] Bernstein P.S., Li B., Vachali P.P., Gorusupudi A., Shyam R., Henriksen B.S., Nolan J.M. (2016). Lutein, zeaxanthin, and meso-zeaxanthin: The basic and clinical science underlying carotenoid-based nutritional interventions against ocular disease. Prog. Retin. Eye Res..

[11] Tudor C., Pintea A. (2020). A Brief Overview of Dietary Zeaxanthin Occurrence and Bioaccessibility. Molecules.

[12] Saini R.K., Nile S.H., Park S.W. (2015). Carotenoids from fruits and vegetables: Chemistry, analysis, occurrence, bioavailability and biological activities. Food Res. Int..

[13] Li B., Gorusupudi A., Arunkumar R., Bernstein P.S. (2022). Extraction, detection, and imaging of the macular carotenoids. Methods Enzymol..

[14] Cui Y., Kong F., Li M., Xu H., Guo Q., Mu Q., Li D., Liu G. (2019). An improved method of simultaneous determination of lutein and zeaxanthin in food. J. Liq. Chromatogr. Relat. Technol..

[15] Stinco C.M., Benítez-González A.M., Meléndez-Martínez A.J., Hernanz D., Vicario I.M. (2019). Simultaneous determination of dietary isoprenoids (carotenoids, chlorophylls and tocopherols) in human faeces by Rapid Resolution Liquid Chromatography. J. Chromatogr. A.

[16] Meléndez-Martínez A.J., Mandić A.I., Bantis F., Böhm V., Borge G.I.A., Brnčić M., Bysted A., Cano M.P., Dias M.G., Elgersma A. (2022). A comprehensive review on carotenoids in foods and feeds: Status quo, applications, patents, and research needs. Crit. Rev. Food Sci. Nutr..

[17] Eriksen J.N., Madsen P.L., Dragsted L.O., Arrigoni E. (2017). Optimized, fast-throughput UHPLC-DAD based method for carotenoid quantification in spinach, serum, chylomicrons, and feces. J. Agric. Food Chem..

[18] Murador D.C., De Souza Mesquita L.M., Neves B.V., Braga A.R., Martins P.L., Zepka L.Q., De Rosso V.V. (2021). Bioaccessibility and cellular uptake by Caco-2 cells of carotenoids and chlorophylls from orange peels: A comparison between conventional and ionic liquid mediated extractions. Food Chem..

[19] Aman R., Biehl J., Carle R., Conrad J., Beifuss U., Schieber A. (2005). Application of HPLC coupled with DAD, APcI-MS and NMR to the analysis of lutein and zeaxanthin stereoisomers in thermally processed vegetables. Food Chem..

[20] Mantzourani I., Nikolaou A., Kourkoutas Y., Alexopoulos A., Dasenaki M., Mastrotheodoraki A., Proestos C., Thomaidis N., Plessas S. (2024). Chemical Profile Characterization of Fruit and Vegetable Juices after Fermentation with Probiotic Strains. Foods.

[21] Meléndez-Martínez A.J., Vicario I.M., Heredia F.J. (2007). Review: Analysis of carotenoids in orange juice. J. Food Compos. Anal..

[22] Amorim-Carrilho K.T., Cepeda A., Fente C., Regal P. (2014). Review of methods for analysis of carotenoids. TrAC Trends Anal. Chem..

[23] Liu H., Zhang Y., Li Q., Zou Y., Shao J., Lan S. (2011). Quantification of lutein and zeaxanthin in marigold (*Tagetes erecta* L.) and poultry feed by ultra-performance liquid chromatography and high performance liquid chromatography. J. Liq. Chromatogr. Relat. Technol..

[24] Bernstein P.S., Khachik F., Carvalho L.S., Muir G.J., Zhao D.Y., Katz N.B. (2001). Identification and quantitation of carotenoids and their metabolites in the tissues of the human eye. Exp. Eye Res..

[25] Aruna G., Mamatha B.S., Baskaran V. (2009). Lutein content of selected Indian vegetables and vegetable oils determined by HPLC. J. Food Compos. Anal..

[26] Junpatiw A., Lertrat K., Lomthaisong K., Tangwongchai R. (2013). Effects of steaming, boiling and frozen storage on carotenoid contents of various sweet corn cultivars. Int. Food Res. J..

[27] Phelan D., Prado-Cabrero A., Nolan J.M. (2018). Analysis of lutein, zeaxanthin, and meso-zeaxanthin in the organs of carotenoid-supplemented chickens. Foods.

[28] Careri M., Elviri L., Mangià A.M. (1999). Liquid Chromatography-Electrospray Mass Spectrometry of b-Carotene and Xanthophylls Validation of the Analytical Method. J. Chromatogr. A.

[29] Mercadante A.Z., Rodrigues D.B., Petry F.C., Mariutti L.R.B. (2017). Carotenoid esters in foods—A review and practical directions on analysis and occurrence. Food Res. Int..

[30] Fratianni A., Mignogna R., Niro S., Panfili G. (2015). Determination of lutein from fruit and vegetables through an alkaline hydrolysis extraction method and HPLC analysis. J. Food Sci..

[31] Rodríguez-Bernaldo de Quirós A., Costa H.S. (2006). Analysis of carotenoids in vegetable and plasma samples: A review. J. Food Compos. Anal..

[32] Phan M.A.T., Bucknall M.P., Arcot J. (2019). Co-ingestion of red cabbage with cherry tomato enhances digestive bioaccessibility of anthocyanins but decreases carotenoid bioaccessibility after simulated in vitro gastro-intestinal digestion. Food Chem..

[33] Phan M.A.T., Bucknall M.P., Arcot J. (2019). Effects on intestinal cellular bioaccessibility of carotenoids and cellular biological activity as a consequence of co-ingestion of anthocyanin- and carotenoid-rich vegetables. Food Chem..

[34] O’Connell O.F., Ryan L., O’Brien N.M. (2007). Xanthophyll carotenoids are more bioaccessible from fruits than dark green vegetables. Nutr. Res..

[35] Montesano D., Gennari O., Seccia S., Albrizio S. (2012). A Simple and Selective Analytical Procedure for the Extraction and Quantification of Lutein from Tomato By-Products by HPLC-DAD. Food Anal. Methods.

[36] Becerra-Moreno A., Alanís-Garza P.A., Mora-Nieves J.L., Mora-Mora J.P., Jacobo-Velázquez D.A. (2014). Kale: An excellent source of vitamin C, pro-vitamin A, lutein and glucosinolates. CYTA-J. Food.

[37] Park S.Y., Choi S.R., Lim S.H., Yeo Y., Kweon S.J., Bae Y.S., Kim K.W., Im K.H., Ahn S.K., Ha S.H. (2014). Identification and quantification of carotenoids in paprika fruits and cabbage, kale, and lettuce leaves. J. Korean Soc. Appl. Biol. Chem..

[38] Carneiro A.M., Lima B.R., Chibli L.A., Carneiro R.L., Funari C.S. (2023). An updated procedure for zeaxanthin and lutein quantification in corn grains based only in water and ethanol. Food Chem..

[39] Murillo E., Meléndez-Martínez A.J., Portugal F. (2010). Screening of vegetables and fruits from Panama for rich sources of lutein and zeaxanthin. Food Chem..

