# Supplementary material for: Quantification of Macular Carotenoids over a Wide Dynamic Range in Plant Matrices and Caco-2 Cells Using a Single Transferable Analytical Method
Source: Foods. 2026 Mar 10;15(6):981. doi: 10.3390/foods15060981 (PMC13025522; doi:10.3390/foods15060981)
Supplement: Supplementary file 1 [file foods-15-00981-s001.zip › foods-4175750-supplementary.pdf]

Supplementary Materials

A.

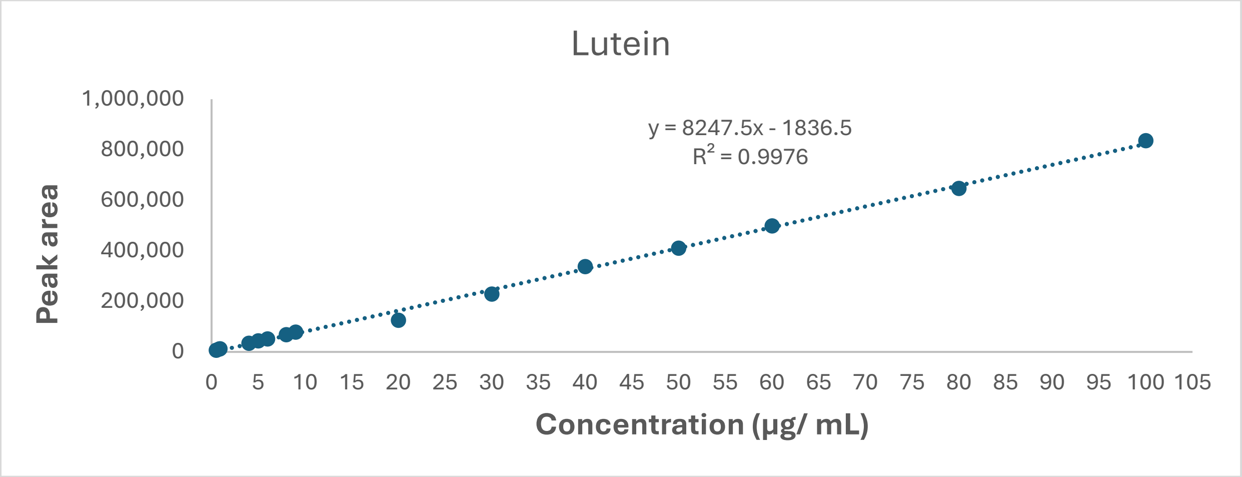

B.

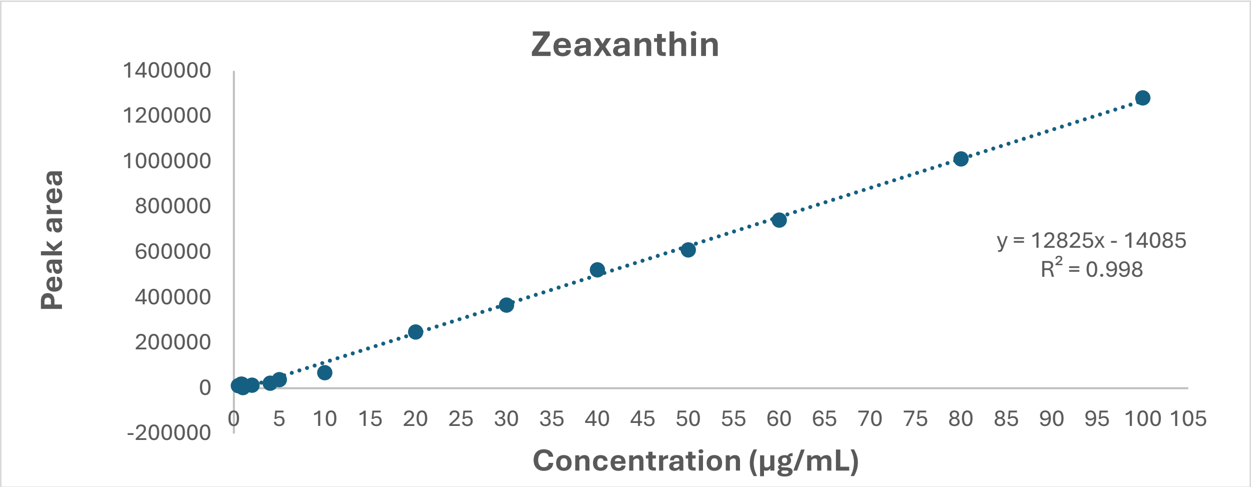

Figure S1: A and B are linearity curves of lutein and zeaxanthin on HPLC, respectively.

C.

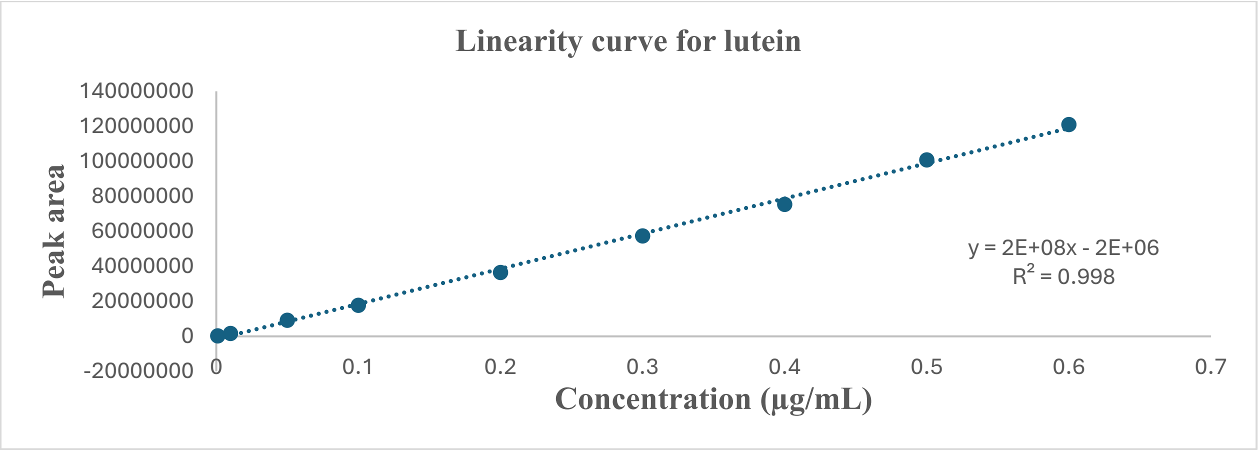

D.

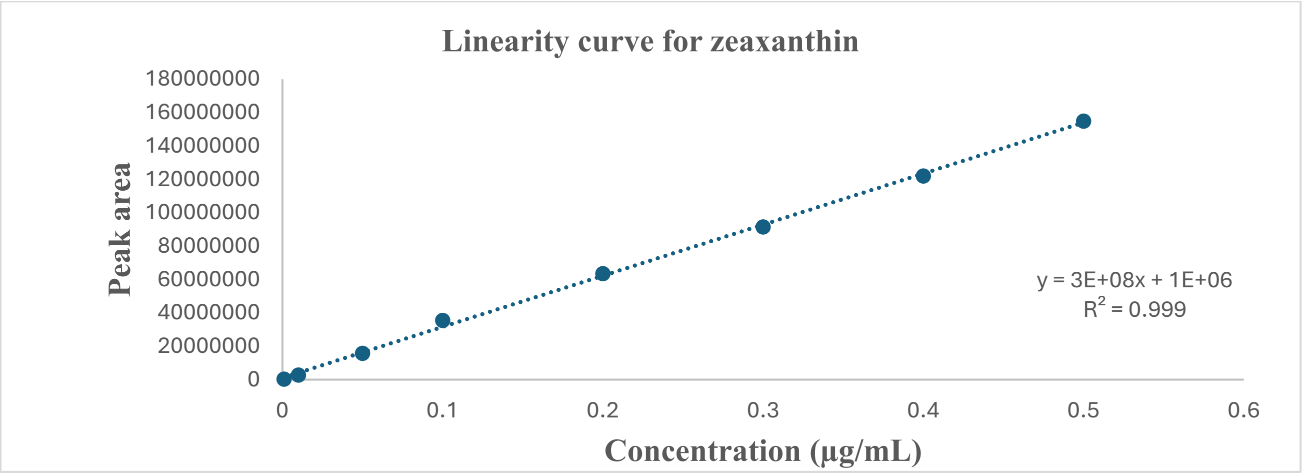

Figure S2: C and D are linearity curves of lutein and zeaxanthin on LC-MS, respectively.

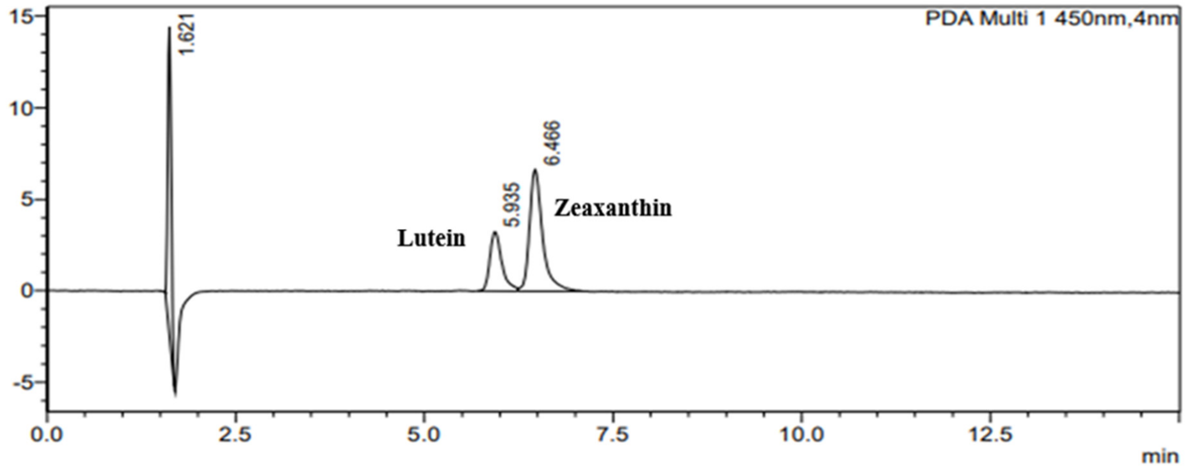

Figure S3: Separation of lutein and zeaxanthin from a mix standard on HPLC.

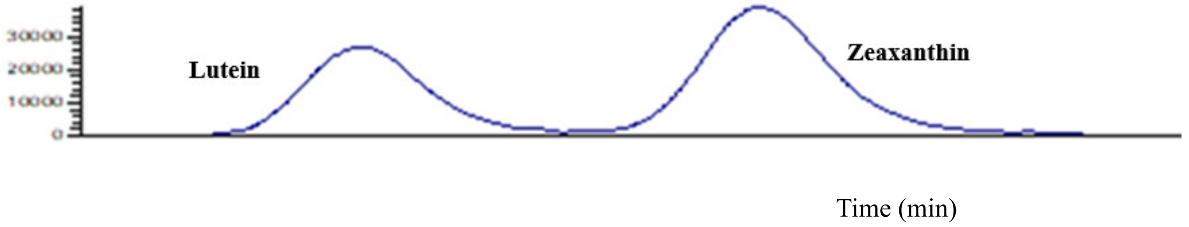

Figure S4: Separation of lutein and zeaxanthin from a mix standard on LC-MS QE Plus

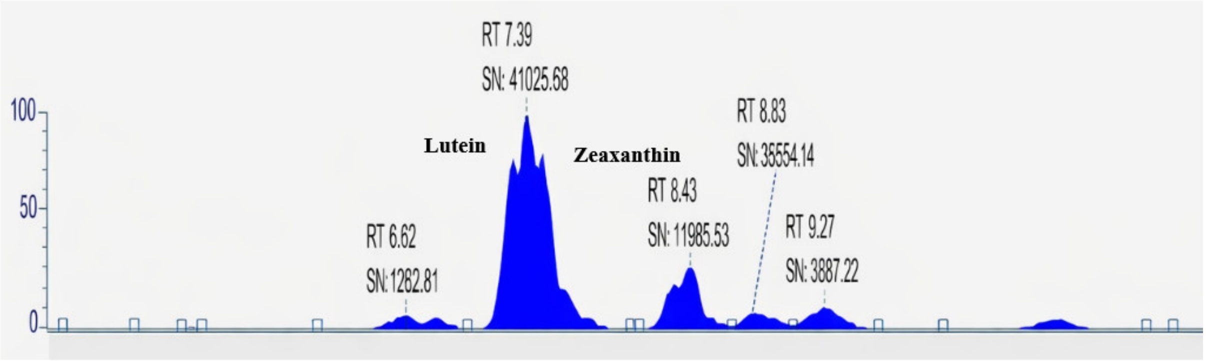

Figure S5: Separation of lutein and zeaxanthin from kale digest on LC-MS QE Plus

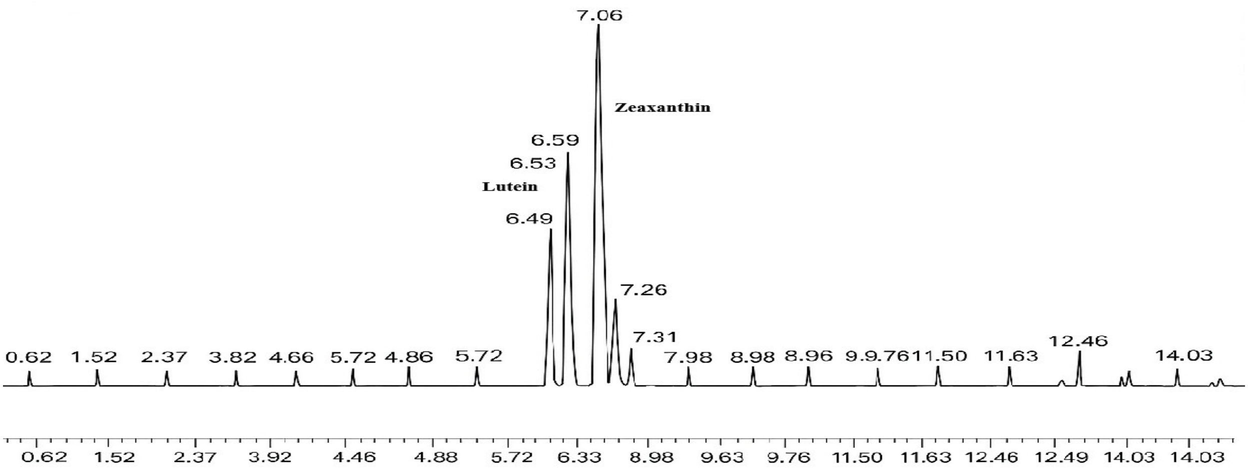

Figure S6: Separation of Lutein and zeaxanthin from kale digesta on LTQ-Orbitrap

A.

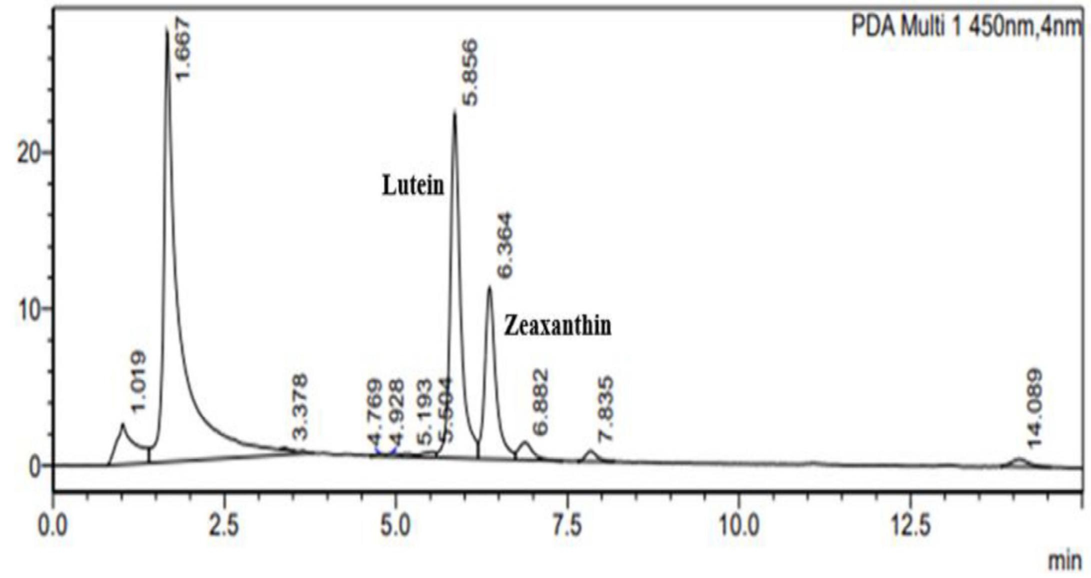

B.

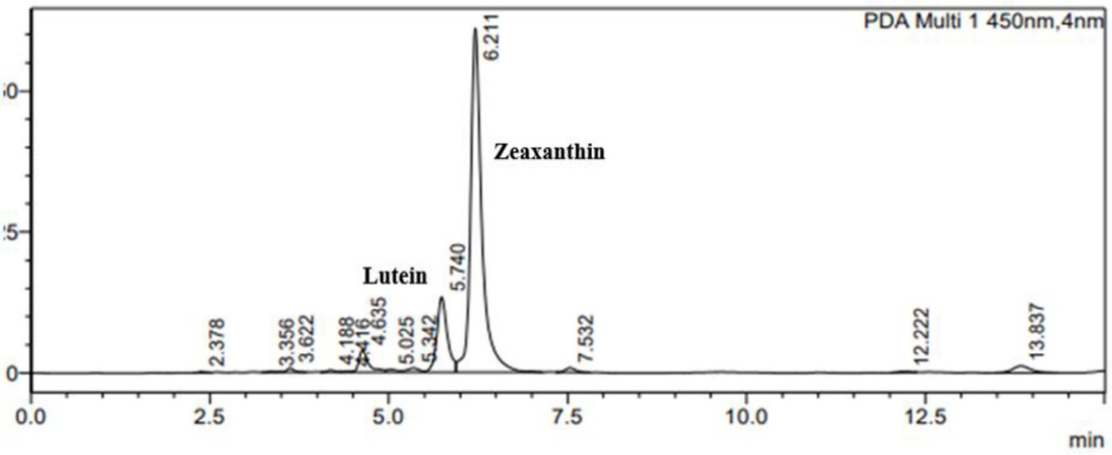

Figure S7. A ; HPLC chromatogram of fresh kale, **B**; HPLC chromatogram of fresh orange pepper.

Table S1. Summary of the generally followed methods reported over the past 10 years for the separation of lutein and zeaxanthin from various samples.

**Sample**	**Column**	**Mobile phase**	**Saponification**	**Reference**
Millet, Corn, Egg, orange, Spinach, Chrysanthemum,	YMC^{TM} C30 (5µm,4.6 250mm)	88% (v/v) methanol solution (containing 0.1% BHT) and methyl tert-butyl ether (MTBE, containing 0.1% BHT)	60% (W/V) KOH for 15 minutes at 50 °C	(Cui et al., 2019)
Human Faeces	YMC C30 (150 × 4.6 mm. 3 µm particle size)	Methanol (solvent A), methyl-tert-butyl ether (solvent B) and water (solvent C)	No saponification was employed	(Stinco et al., 2019)
Fruits and vegetables	**Normal phase** conditions, using a 250 × 4.6 mm 5µm particle size,	n-hexane/isopropyl alcohol (5%)	Alkaline hydrolysis was employed with KOH, NaCl and ethanol	(Fratianni et al., 2015)
Orange Peel	HPLC-PDA-MS/MS<br><br>C30 YMC column (5 µm, 250 × 4.6 mm)	A (methanol/MTBE/ H2O, 81:15:4) and B (methanol/MTBE/H2O, 16:80:4),	Extraction with ionic liquid ; 1-butyl-3-methylimidazolium chloride ([C4mim]Cl)	(Murador et al., 2021)
Fruit and vegetable juices	UPLC-QtoF/MS<br><br>C18column(2.1×100mm,2.2µm)	A - water/methanol (90:10 v/v,)B -methanol (both containing 5 mM of ammonium acetate	The extraction method was not specified	(Mantzourani et al., 2024)
Corn grains	UHPLC-PAD<br><br>C18 150 × 3.0 mm, 1.8 µm	water (A) and ethanol (B),	No saponification was employed	(Carneiro et al., 2023)

Chilli pepper	HPLC	acetone (solvent A) and water (solvent B)	Saponification employed	(Xu et al., 2023)
Spinach<br><br>Serum, Chylomicrons<br><br>Faeces	UHPLC-DAD<br><br>C18 (1.8 µm, 2.1 × 150 mm)	75% acetonitrile/dichloromethane/ methanol (75:10:15) (B) and acetonitrile/dichloromethane/methanol (60:10:30) (A).	No saponification was employed	(Eriksen et al., 2017)
Fruits and vegetables	HPLC<br><br>Reverse- phase C18 (250 x 4.6mm)	acetonitrile-methanol-dichloromethane (75:20:5)	No saponification was employed	(O'Connell et al., 2007)
Costa Rican Acrocomia aculeata fruits	HPLC-DAD-APCI/ESI-MS^n<br><br>C30 reverse- phase column (250 ×4.6mm i.d., 3µm particle size,	methanol/tBME/water (80:18:2, v/v/v, eluent A; 8:90:2, v/v/v, eluent B) containing 0.4 g/L ammonium acetate.	No saponification was employed	(Schex et al., 2018)

Table S2: The LOD and LOQ values from the previously validated methods to separate lutein and zeaxanthin.

Instrument	Lutein		Zeaxanthin		Reference
	LOD	LOQ	LOD	LOQ	
UHPLC	0.02mg/100g	0.1mg/100g	0.04mg/100g	0.2mg/100g	(Liu et al., 2011)
UHPLC	3µg/100g	9µg/100g	3µg/100g	9µg/100g	(Cui et al., 2019)
HPLC	0.020 mg/L	0.063 mg/L	0.067mg/L	0.209mg/L	(Xu et al., 2023)
UHPLC	0.0038 µg/mL	0.0618 µg/mL	0.0038 µg/mL	0.0618 µg/mL	(Carneiro et al., 2023)
**HPLC**	**0.03 µg/mL**	**0.12 µg/mL**	**0.0 3µg/mL**	**0.12 µg/mL**	**Present method**
**LC-MS APCI negative mode**	**0.40 ng/mL**	**2.10 ng/mL**	**0.40 ng/mL**	**2.10 ng/mL**	

Table S3.

HPLC Instrumental precision (seven consecutive injections of the same extract)

Injection of an extract (Replicates n◦)	Lutein		Zeaxanthin	
	Peak area	Rt (min)	Peak area	Rt (min)
1	5278719	5.77	589901	6.22
2	5278689	5.77	589809	6.22
3	5276987	5.72	589699	6.22
4	5198992	5.75	589810	6.21
5	5269897	5.76	589724	6.20
6	5213456	5.78	589790	6.21
7	5275485	5.77	589802	6.21
Mean	5252790	5.754	589778.8	6.21
Standard deviation (SD)	33323.17	0.018	65.3549	0.007
Relative standard deviation (RSD -%)	0.60	0.30	0.10	0.10

Table S4.

LC - MS Instrumental precision (Seven consecutive injections of the same extract)

Injection of an extract (Replicates n◦)	Lutein		Zeaxanthin	
	Peak area	Rt (min)	Peak area	Rt (min)
1	3734500	5.510	5985225	5.946
2	3629864	5.510	5945879	5.960
3	3634567	5.509	6010095	5.858
4	3689008	5.508	6000876	5.950
5	3700285	5.509	5967874	5.949
6	3667805	5.510	5985225	5.946
7	3647850	5.511	6045379	5.960
Mean	3671983	5.51	5991508	5.94
Standard deviation (SD)	35401.88	0.001	29410.5	0.03
Relative standard deviation (RSD -%)	0.96	0.01	0.50	0.50

Table S5.

Intra-day and inter day precision (5 extracts were prepared and injected for 3 days) on HPLC

**Extraction n◦**	**Days of analysis**	**Lutein**		**Zeaxanthin**	
		**Peak area**	**Retention time (min)**	**Peak area**	**Retention time (min)**
1	1	7802	5.895	4462	6.427
2	1	7840	5.915	4365	6.443
3	1	7715	5.895	4473	6.410
4	1	7790	5.892	4230	6.435
5	1	7810	5.800	4550	6.410
Mean day 1		7791.4	5.8794	4416	6.425
SD day 1		41.62	0.04	110.02	0.0132
RSD (%) day 1		0.005	0.006	0.025	0.002
1	2	7876	5.809	4591	6.31
2	2	7777	5.764	4505	6.269
3	2	7631	5.819	4574	6.288
4	2	7632	5.815	4565	6.269
5	2	7940	5.795	4612	6.268
Mean day 2		7771.2	5.802	4569.4	6.281
SD day 2		125.33	0.02	35.97	0.016
RSD (%) day 2		0.016	0.003	0.008	0.003
1	3	7580	5.815	4198	6.318
2	3	7824	5.840	4640	6.351
3	3	7727	5.866	4747	6.408
4	3	7878	5.815	4759	6.387
5	3	7980	5.892	4791	6.318
Mean day 3		7797.8	5.846	4627	6.3564
SD day 3		136.209	0.03	220.42	0.036
RSD (%) day 3		0.017	0.005	0.047	0.006
Mean day 1-3		7786.8	5.841	4537.47	6.354
SD day 1-3		11.34	0.04	89.05	0.07
RSD (%) day 1-3		0.001	0.006	0.02	0.01

Table S6.

Intra-day and inter day precision (5 extracts were prepared and injected for 3 days) on LC-MS

**Extraction n◦**	**Days of analysis**	**Lutein**		**Zeaxanthin**	
		**Peak area**	**Retention time (min)**	**Peak area**	**Retention time (min)**
1	1	3835449	5.509	5985225	5.946
2	1	3855662	5.509	5945879	5.960
3	1	3792980	5.449	6010095	5.858
4	1	3789990	5.510	5899876	5.950
5	1	3847881	5.510	5967874	5.949
Mean day 1		3824392.4	5.4974	5961790	5.9326
SD day 1		27647.84	0.024	37432.85	0.038
RSD (%) day 1		0.007	0.004	0.006	0.006
1	2	3628794	5.559	5945233	6.10
2	2	3549865	5.480	5788970	6.12
3	2	3652111	5.514	5912451	5.567
4	2	3698768	5.486	5898780	5.943
5	2	3574689	5.504	5857942	5.965
Mean day 2		3620845.4	5.509	5880675	9.94
SD day 2		53443.95	0.028	53724.92	0.198
RSD (%) day 2		0.014	0.005	0.009	0.033
1	3	3734500	5.456	5878654	5.89
2	3	3629864	5.456	5849890	5.89
3	3	3534567	5.390	5765423	5.92
4	3	368900	5.445	5723516	5.90
5	3	3700285	5.445	5825982	5.86
Mean day 3		3657663.2	5.4384	5808693	5.892
SD day 3		70191.53	0.024	56600.46	0.019
RSD (%) day 3		0.019	0.004	0.010	0.003
Mean day 1-3		3700967	5.481467	5883719	5.9212
SD day 1-3		88559.81	0.031	62538.56	0.019
RSD (%) day 1-3		0.023	0.006	0.011	0.021

Table S7: Results of spike samples of kale on HPLC

	Initial content (µg/g DM)	Added value (µg/g)	Results (µg/g DM)	Recovery (%)	RSD (%)
Lutein R1	420.70	100	514.45	98.80	0.06
R2	412.20	100	506.64	98.91	
R3	407.86	100	508.48	100.12	
Zeaxanthin R1	34.50	100	130.74	97.20	0.82
R2	32.38	100	131.02	98.97	
R3	30.95	100	127.32	97.23	

*R1, R2, R3 are replicates

Table S8: Results of spike samples of Caco-2 cell extracts on LC-MS

	Initial content (µg/g DM)	Added value (µg/g)	Results (µg/g DM)	Recovery (%)	RSD (%)
Lutein R1	1.09	1	1.90	90.91	1.79
R2	0.95	1	1.82	93.33	
R3	0.88	1	1.69	89.36	
Zeaxanthin R1	0.74	1	1.58	90.80	
R2	0.78	1	1.60	89.88	
R3	0.65	1	1.51	91.52	

*R1, R2, R3 are replicates
